# Donor/recipient origin of lung cancer after lung transplantation by DNA short tandem repeat analysis

**DOI:** 10.3389/fonc.2023.1225538

**Published:** 2023-09-28

**Authors:** Julien De Wolf, Edouard Robin, Alexandre Vallee, Justine Cohen, Abdul Hamid, Antoine Roux, Morgan Leguen, Romane Beaurepere, Ivan Bieche, Julien Masliah-Planchon, Matthieu Glorion, Yves Allory, Edouard Sage, the Foch lung transplant group

**Affiliations:** ^1^ Department of Thoracic Surgery and Lung Transplantation, Foch Hospital, Suresnes, France; ^2^ Department of Clinical Research and Innovation Foch Hospital, Suresnes, France; ^3^ Department of Anatomopathology, Foch Hospital, Suresnes, France; ^4^ Department of Pneumology, Foch Hospital, Suresnes, France; ^5^ Department of Anesthesiology, Foch Hospital, Suresnes, France; ^6^ Université Paris-Saclay, INRAE, UVSQ, VIM, Jouy-en-Josas, France; ^7^ Genetics Department, Curie Institut, Paris, France; ^8^ Department of Anatomopathology, Curie Institut, Paris, France

**Keywords:** lung transplantation (LT), short tandem repeat (STR), desoxyribonucleic acid (DNA), polymorphism chain reaction (PCR) transplantation, lung cancer, chimerism, STR, donor

## Abstract

**Background:**

Lung cancer is more common in posttransplant recipients than in the general population. The objective of this study was to examine the chimerism donor/recipient cell origin of graft cancer in recipients of lung transplant.

**Methods:**

A retrospective chart review was conducted at Foch Hospital for all lung transplantations from 1989 to 2020. Short tandem repeat PCR (STR-PCR) analysis, the gold standard technique for chimerism quantification, was used to determine the donor/recipient cell origin of lung cancers in transplant patients.

**Results:**

Fourteen (1.4%) of the 1,026 patients were found to have graft lung cancer after lung transplantation, and one developed two different lung tumors in the same lobe. Among the 15 lung tumors, 10 (67%) presented with adenocarcinoma, four (27%) with squamous cell carcinoma and one with small cell lung cancer. STR analysis showed that the origin of the cancer was the donor in 10 patients (71%), the recipient in three patients (21%), and was undetermined in one patient. Median time to diagnosis was 62 months.

**Conclusion:**

The prevalence of lung cancer in lung transplant recipients is very low. However, the results of our study showed heterogeneity of genetic alterations, with 21% being of recipient origin. Our results highlight the importance of donor selection and medical supervision after lung transplantation.

## Introduction

Lung transplantation provides a life-saving solution for patients with end-stage pulmonary diseases, including idiopathic pulmonary fibrosis, chronic obstructive pulmonary disease, and cystic fibrosis, which are common indications for lung transplantation. Initially considered a rare extreme surgical intervention, lung transplantation is now an established therapeutic strategy for selected patients and has greatly improved since the first lung transplantation in 1983 (1). This surgical procedure is now commonplace, with acceptable short- and intermediate-term survival. An increased number of patients achieve long-term survival, with a median survival of approximately seven years (2).

As outcomes have improved, chronic medical illnesses have emerged as another main obstacle to long-term survival. In recent years, the prevalence of lung cancer in lung transplant recipients has increased (3, 4). The International Society for Heart and Lung Transplantation (ISHLT) has reported that cancer is the second most common cause of death in lung transplant recipients 5 to 10 years after transplantation (17.3%) and in those with more than 10 years after transplantation (17.9%) (2, 5). Among lung transplant recipients, lung cancer is the second most common cancer after skin cancers, but above post-transplant lymphoproliferative disorder (6).

Cancers after solid organ transplantation mainly develop *de novo* in recipient (7). Pretransplant malignancies may be the cause of cancer development in recipients. The literature has rarely donor-related tumors (8). Donor-related pathology could arise as a result of cancer transmission from a previously known or unknown malignancy in the donor or as malignant transformation of donor cells within the recipient without a previous malignancy (9, 10).

Nevertheless, there are only a few studies showing data on the origin of malignancy to be a tumor after lung transplant, while the increased risk of cancer post-lung transplant has been documented (11, 12). A better understanding of the origin of lung cancer after lung transplantation could help identify methods to improve transplant safety and prognoses for patients who develop lung cancer. The coexistence of cells of different genetic origins within the same organism is a biological chimera (13). Assessment of the proportion of DNA belonging to the recipient or donor after transplantation is called chimerism analysis (14). A situation in which only donor DNA is detected in a post-transplant sample is called complete chimerism, whereas the detection of both donor and recipient DNA is referred to as mixed chimerism. Several biomarkers have been used to quantify chimerism. Currently, the gold standard technique is short tandem repeat PCR (STR-PCR) (15, 16). STR, also known as microsatellites, is a genomic DNA sequence consisting of repeated units of two- to six base pairs (17). PCR-STR is the gold standard for chimerism quantification and evaluation. In eukaryotic genomes, STRs are extensively distributed in non-coding regions and are characterized by co-dominant inheritance, high polymorphism levels, high reliability, and good reproducibility, thus making them effective for forensic analysis (17, 18). As second-generation genetic markers, STRs are broadly used in forensics for personal identification and paternity testing (18).

Therefore, STR can be used to identify donor/recipient origin of graft cancer cells in post-transplant patients. To this end, we conducted a retrospective study of 14 cases of post-transplant graft lung cancer at Foch Hospital.

## Methods

We conducted a retrospective chart review of all patients who underwent lung transplantation at the Foch Hospital between 1989 and 2020. Cancer diagnosis was defined using anatomopathological biopsies. The study was reviewed and approved by Foch Hospital institutional review board IRB00012437 (Protocol Number 20-04-08). Written informed consent was obtained from all participants [individual(s) AND/OR minor(s)’ legal guardian/next of kin] for the publication of any potentially identifiable images or data included in this article.

During the study period, 1,026 subjects underwent lung transplantation at the Foch Hospital. Of these, 26 (2.6%) patients developed lung cancer after lung transplantation.

**Figure 1:**
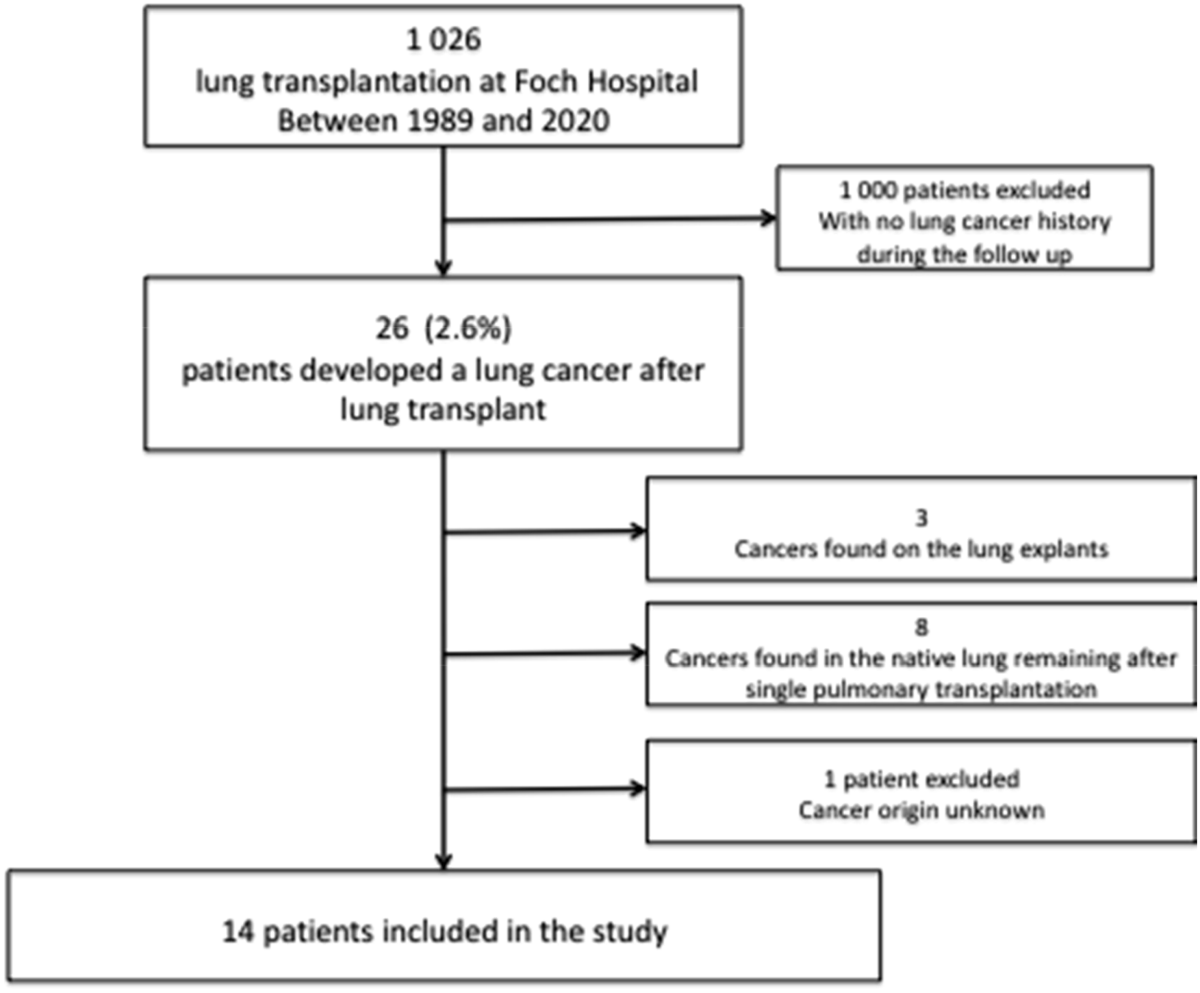
Study flowchart.

Cancers found in the lung explants of recipients were excluded (N = 3), as well as cancers found in the native lung remaining after single pulmonary transplantation (N = 8) and patients presenting with cancer of unknown primary origin (N = 1). Ultimately, 14 patients with cancer found in the transplanted lung were included in the analysis, and two of them, two tumors (one lung and one brain tumor), were analyzed (**Figure 1**).

### STR analysis

Three separate tissues were analyzed. The first, corresponding to the recipient, was from the explanted lungs; the second was from the graft itself, either via transbronchial biopsies or via lung samples taken in the operating room on the graft; and the third tissue was the anatomopathological piece for the diagnosis of cancer or the surgical excision piece if curative surgery had been performed.

STR analysis was performed using PCR, and the PCR products was detected using a 3500XL Genetic Analyzer (Applied Biosystems®) with the AmpFℓSTR™ Identifiler™ Plus PCR Amplification Kit (Applied Biosystems®). Using STR-PCR technology, we observed some tumors with, as expected, cellular chimerism. Tumors were of recipient origin when only the recipient origin DNA was detected in the tumor (i.e., absolutely no donor DNA was detected) **(** **Supplementary File 1** **)**.

### FISH analysis

Four patients had a sex mismatch with donor lung grafts. We confirmed the tumor origin results evidenced by STR using fluorescent *in situ* hybridization (FISH) targeting the sexual Y chromosome. The Y chromosome in the tumor and surrounding normal lung graft tissue was examined. FISH for *Chromosome Y* gene detection was performed as follows: First, 3 µm-thick FFPE tissue slides were dewaxed, dehydrated, cooked for 12 min in a pretreatment buffer at 80°C (Abbott Molecular, IL, USA), and then treated with protease solution (Abbott Molecular, IL, USA) for 14 min at 37°C. Hybridization was then performed with SureFISH ChrY CEP 273kb RD (Agilent Technologies, Santa Clara, California, USA), at 82°C for 5 min and then at 37°C for 22 h in a ThermoBrite Statspin Hybridizer (Abbott Molecular, IL, USA). Non-specific binding was removed by immerging the slides in a wash buffer I (0.7XSSC, 0.3% NP40 pH 7) 10 min at room temperature then in the wash buffer II (2XSSC, 0.1% NP40 pH 7) at 65°C 5 min. The slides were then rinsed three times in distilled water, dehydrated, air-dried, and mounted with VECTASHIELD Antifade Mounting Medium with DAPI (Clinisciences, Nanterre, France) and a cover glass. The samples were stored at 4°C until further use.

### Histopathological confirmation of the correct origin characterization

To ensure that the tumor from the recipient origin was correctly characterized, we checked the necrosis and tumor cell percentage for each case. We observed no necrosis and a percentage of tumor cells for the three tumors of 60%, 50%, and 20%, respectively.

### Data analysis

The characteristics of the study population were described as mean and standard deviation (SD) for continuous variables. Categorical variables are described as absolute numeric values and proportions. Statistical analyses were performed using the SAS software (version 9.4; SAS Institute, Cary, NC, USA).

## Results

The mean age of the recipients was 39.8 (11.6) years. Of the 1,026 transplanted patients with unilateral or bilateral pulmonary tumors, only 14 presented lung cancer on the graft (1.4%), and one of these patients developed two different lung tumors in the same lobe. Lung cancer was detected in the pulmonary transplant patients and was uniformly distributed throughout the study period. Of the 14 patients, 12 had bilateral lung transplants. The three causes for lung transplantation were cystic fibrosis in eight patients, emphysema in two, fibrosis in three, and lymphangiomatosis in one (**Table 1**). Only four (28%) transplanted patients presented a sex mismatch with the donor (**Table 1**).

**Table 1:** Characteristics of the study population.

Recipient age, mean (SD)	39.8 (11.6)
Women, no. (%)	6 (42.9)
Smokers no. (%)	6 (60.0)
Bilateral lung transplant, no. (%)	12 (85.7)
Diagnosis, no. (%)	
Cystic fibrosis	8 (57.1)
Emphysema	2 (14.3)
Pulmonary fibrosis	3 (21.4)
Lymphangiomatosis	1 (7.2)
Lung cancer, no. (%)	
Adenocarcinoma	10 (67)
Squamous cell carcinoma	4 (27)
Small cell lung cancer	1 (6)
Time to cancer discovery, mean (SD)	75.2 (47.1)
Donor age, mean (SD)	51.7 (10.8)
Women donor, no. (%)	6 (42.9)
Tobacco smoking donor, no. (%)	5 (46.2)
Sex mismatch, no. (%)	4 (28.6)

Of the 14 transplanted patients (15 lung tumors examined), 10 presented with invasive adenocarcinoma (67%), four with squamous cell carcinoma (27%), and one with small cell lung cancer (6%). The time to the discovery of lung cancer in the 14 subjects ranged from 12 to 160 months post-transplantation, with a median of 63 months (**Table 1**). The characteristics of the 15 lung cancer cases with donor and recipient information are presented in **Table 2**.

**Table 2:** Characteristics of the lung cancers with donor and recipient information.

	Recipient			Donor		Lung Cancer		Origin of cancer	Cancer treatment
	Age (year)	Tobacco history (1Yes, 0 No)	Indication for Lung Transplantation	Age (year)	Tobacco history (1Yes, 0 No)	Histological type	Delay of occurrence after LT (months)		
1	42	0	Lymphangiomyomatosis	51	UN	Adenocarcinoma	114	NI	None
2	55	0	Hypersensitivity pneumonia	47	0	Epidermoid carcinoma	37	Recipient	Chemotherapy
3	52	1	Emphysema	48	1	Adenocarcinoma	64	Donor	Chemotherapy
4	33	0	Cystic fibrosis	63	0	Adenocarcinoma	48	Donor	Lobectomy + Chemotherapy
5	57	1	Fibrosis	35	0	Epidermoid carcinoma	61	Recipient	Chemotherapy + Radiotherapy
6	25	0	Cystic fibrosis	55	0	Small cell lung cancer	12	Donor	Lobectomy
7	49	0	Cystic fibrosis	46	1	Adenocarcinoma	50	Donor	Lobectomy
8	48	1	Emphysema	41	1	Adenocarcinoma	120	Donor	Segmentectomy
9	24	0	Cystic fibrosis	56	0	Epidermoid carcinoma	148	Donor	Chemotherapy
10	31	0	Cystic fibrosis	70	0	Adenocarcinoma	87	Donor	Lobectomy
11	29	0	Cystic fibrosis	60	1	Adenocarcinoma	24	Donor	Segmentectomy
12	51	1	Fibrosis	55	1	Adenocarcinoma	31	Recipient	Chemotherapy
13	33	0	Cystic fibrosis	56	0	Adenocarcinoma	84	Donor	Lobectomy
14	34	0	Cystic fibrosis	43	1	Adenocarcinoma	160	Donor	Lobectomy + Chemotherapy
						Epidermoid carcinoma	160	Donor	

UN, unknown; NI, non-interpretable.

**Figure 2:**
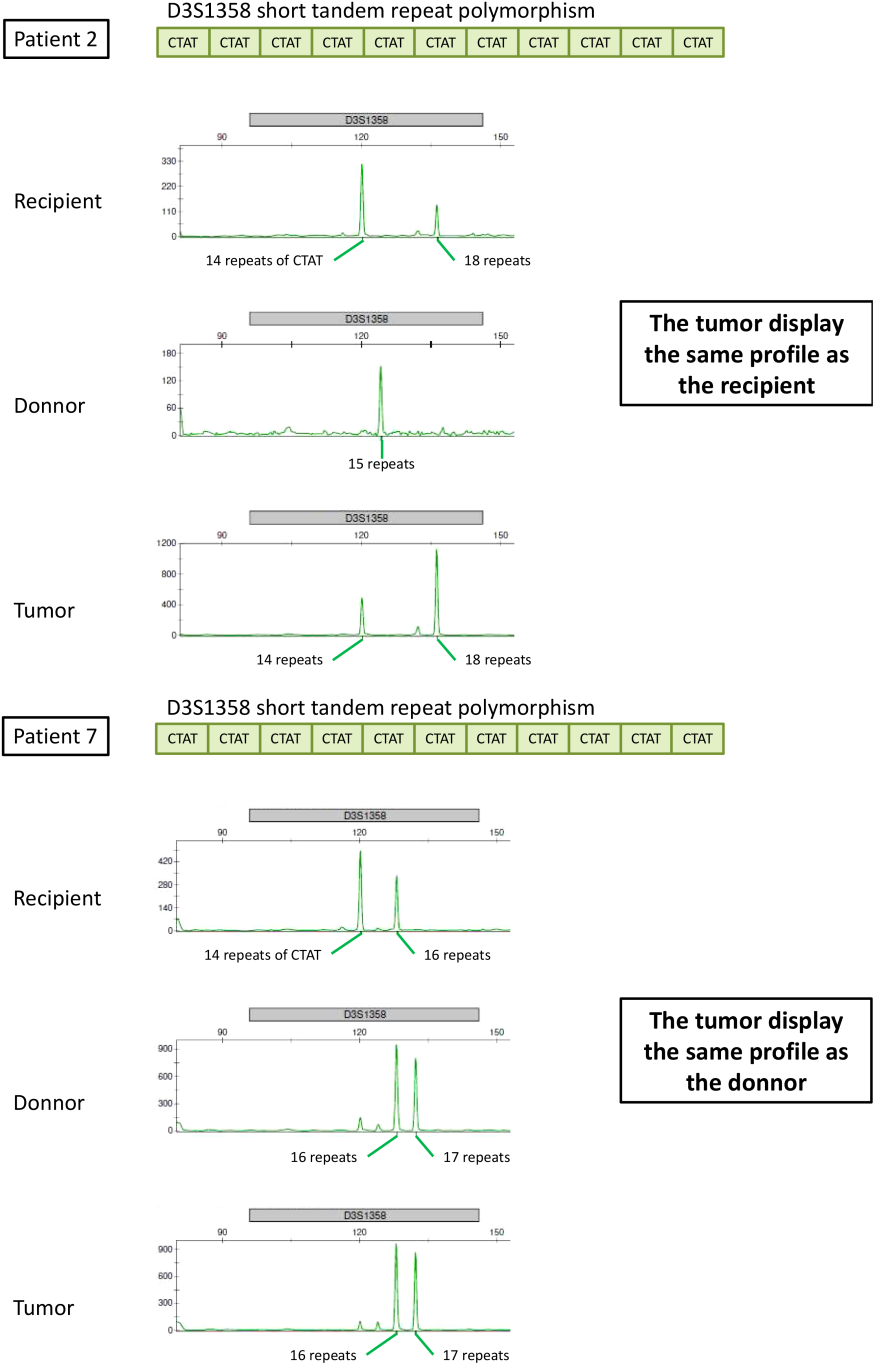
STR analysis interpretation for donor origin and recipient origin.

STR analysis showed that the origin of the cancer was the donor in 10 patients (71%), the recipient in three patients (21%), and was undetermined in one patient (**Table 3** and **Figure 2**). Six patients presented with a lack of information or tissue contamination, i.e., four patients with a lack of informative STR analysis and two patients with tumor and donor tissues contaminated by the recipient (**Table 3**).

**Table 3:** STR analysis results for the post-transplant patients.

Patients	Cancer origin	Sex recipient Amelogenin	Sex donor Amelogenin	Sex cancer Amelogenin	Remarks
1	NI	NI	NI	NI	
2	Recipient	Male	NI	Male	
3	Donor	Male	NI	Male	Few informative STR
4	Donor	Male	Female	Female	
5	Recipient	NI	Female	Female	
6	Donor	Female	Female	Female	Few informative STR
7	Donor	Female	Male	Male	
8	Donor	Female	Male	Male	
9	Donor	Female	Female	Female	Cancer difficult to interpret
10	Donor	Female	Female	Female	
11	Donor	Male	Male	Male	
12	Recipient	NI	Male	Male	Recipient almost not interpretable
13	Donor	Male	Male	Male	Tumor and donor contaminated by the recipient
14	Donor	Female	Female	Female	Tumor and donor contaminated by the recipient two tumors were found in the left upper lobe (Adenocarcinoma and Epidermoid carcinoma)
	Donor	Female	Female	Female	

NI, not informative.

In addition to amelogenin, the percentage of informative loci was 27% for STR-PCR, which is concordant with previous publications (19, 20), with more than 12 patients explained by D8S1179 (13 patients), D3S1358 (13 patients), D19S433 (13 patients), and TH01 (12 patients) (**Supplementary Table 2**).

**Figure 3:**
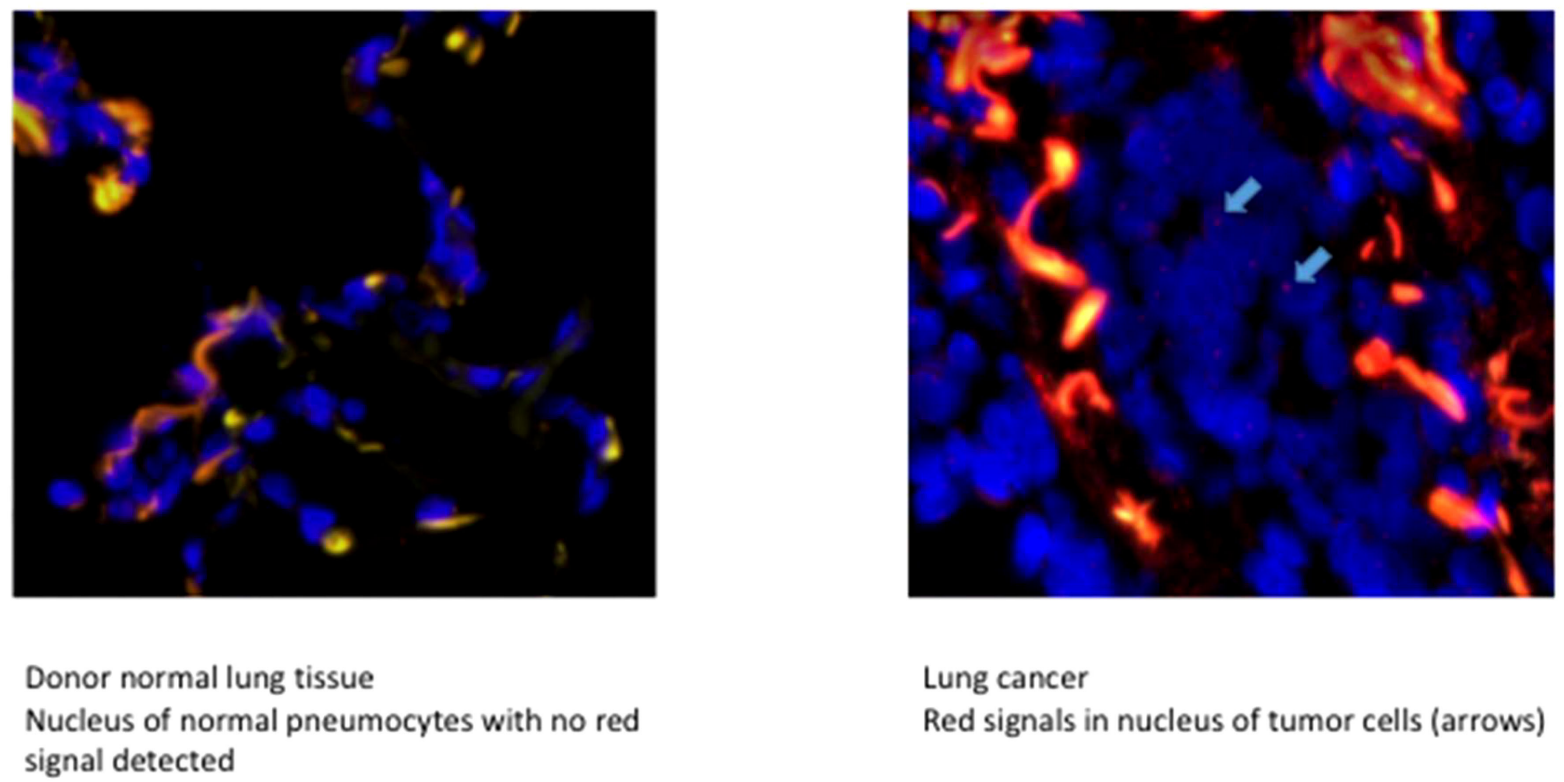
FISH of the patient with a tumor receiver origin on a sex mismatch Donor/Receiver.

Four patients were mismatched and evaluated using FISH targeting the Y-chromosome. Of these patients, only one had the recipient origin of his cancer through the STR technique. The FISH results matched perfectly with the previously described technique and were clearly confirmed (**Figure 3**).

At the end of data collection, seven patients died (50%). The mean time to cancer diagnosis after LT was 74 months. Death occurred an average of 21.4 months after cancer diagnosis.

In the donor population, the mean age at transplantation time was 35.8 years, and the time to cancer diagnosis after LT was 79 months. All patients with a donor origin underwent cancer treatment, and eight of them underwent surgical management. Four patients received chemotherapy, two received adjuvant therapy, and two received isolated systemic therapy. None of the patients received targeted therapy or immunotherapy (**Table 2**).

Regarding the three cancers where the origin was the recipient, they were all older than 50 years, all were male, transplanted for pulmonary fibrosis, and had a history of smoking. The mean time to cancer diagnosis after LT was 43 months. These three patients were diagnosed at a disseminated stage of neoplasia; one of them had no tumor images in the lung parenchyma, but only in the mediastinal lymph nodes and brain. None of the patients received surgical treatment, and the average survival after cancer diagnosis was 7.1 months (**Table 2**).

## Discussion

Lung cancer is more common in post-transplant recipients than in the general population (6, 21). Lung cancer risk was elevated 5-fold in lung recipients compared that to in the general population (22, 23). Moreover, the recent US Scientific Registry for Transplant Report (SRTR) indicates a rate 13 times higher in lung transplant recipients than in the general population (6, 21) with prevalence rates ranging from 1% to 9% (7, 8, 24). However, the main indications for lung transplantation appear to be independent of the risk of developing lung cancer (5).

Learning whether the newly diagnosed cancer that occurred after transplantation came from the native or transplanted organ could reduce the risk of similar issues in future transplant patients. Recipient origin could be a major source of information for future transplant patients because we found that 21% of graft lung cancers in our study had a recipient origin. Moreover, even in cases of double transplantation, it cannot be assumed that the cancer could only originate from donor cells because of the possibility of cellular epithelial chimerism that may persist years after transplantation as well as residual tracheobronchial tissue after surgery (25). However, when the time between transplantation and cancer diagnosis appears short and the test determines donor origin, it may be fair to conclude that the malignancy pre-existed in the donor and was therefore transmitted to the recipient. However, if the time between transplanted patients and diagnosis is several years and the test determines donor origin, cancer may have developed *in situ* due to prolonged immunosuppression in relation to donor and recipient risk factors (26).

Lung cancer can be observed in four clinical situations after lung transplantation: (a) lung cancer in the native lung after lung transplantation, (b) detection of lung cancer in the explanted diseased lung, (c) lung cancer growth in the allograft (donor transmitted or *de novo* malignancy), and (d) recurrence in a patient transplanted for the primary indication of lung cancer.

### Biologic chimera

The main cases of lung cancer observed in lung transplant recipients occur in their natal lungs. According to the literature, most lung cancers (certainly in the remaining native lung) originate from the recipient (27). De Soyza et al. described the donor origin of lung cancer (28). Another report showed that a patient with cystic fibrosis who underwent a double lung transplant developed small cell lung cancer. However, when the donor was male, the tumor was found to have a female origin (29). These cases report the donor origin of bronchial chimerism after lung transplantation. It has been shown that endothelial cells and bronchial and alveolar epithelium retain the donor sex type while donor lymphocytes are gradually replaced by recipient cells (30). Similarly, the integration of recipient-derived cells has been observed in the bronchial epithelium, type II pneumocytes, and seromucous glands. Epithelial structures that mimic chronic injury have a higher degree of chimerism than other structures (31).

The particular case of children well describes this process, following sex mismatched lung transplantation, transbronchial biopsies have shown the onset of the chimerism process as early as 3 weeks post-transplantation, and this process remains constant up to 37 months later (25). Thus, the hypothesis is that host stem cells or progenitor cells replace lung cells in the transplanted lung, which are lost as a result of various processes affected in lung transplantation (25).

### Recipient transmitted malignancies

In the general population, exposure to carcinogenic toxins (e.g., tobacco smoke), advanced age, and male sex are associated with an increased risk of lung cancer after transplantation. It is important to note that generally 20% of transplants since the early 2000s have involved recipients over the age of 59 years (32). Surgical procedures necessarily expose a native lung exposed to these conditions (6, 22). Therefore, the donor lung is the most likely cause of lung cancer. This state of development in the native lung represents 12 of the 23 cases reported by the Madrid team in 2017 (22); nine of the 13 cases reported by the Leuven team in 2012 (33) or eight of the nine cases reported by the Cordoba team in 2012 (34).

Terminal respiratory diseases eligible for transplantation and lung cancer have common environmental risk factors such as smoking, asbestos, or silica exposure (7). In addition, interstitial lung diseases, such as idiopathic pulmonary fibrosis, have molecular pathogenic pathways similar to carcinogenesis (35). Immunosuppressive treatment used for organ transplants can generate virus-induced malignancy and inhibit the immune antitumor response (36).

Immunosuppressive drugs, such as calcineurin inhibitors (INNs), cyclosporin and tacrolimus, can directly act on carcinogenesis and improve tumor progression by inhibiting DNA repair (37), inhibiting the apoptotic action of damaged cells (38), and promoting cell and metastatic migration and progression (39). However, new immunosuppressive drugs, such as mycophenolate mofetil (MMF) and mechanistic target of rapamycin (mTOR), can reduce tumor growth (40).

### Donor transmitted malignancies

The latest registries show increased use of smoking and advanced donor lungs, with the lung share of donors over 55 years of age doubling between 2000 and 2009, according to the data of the United Network for Organ Sharing (41).

However, lung cancer in allografts after lung transplantation is still rare because of a careful donor selection mechanism (42). However, faced with organ shortages, transplant centers increasingly use older donors or donors with a history of smoking. Currently, few data are available on the possible impacts, including the incidence of lung cancer in allografts (8, 23, 42, 43).

Recipient pre-transplant screening with chest CT-scan has made tumor detection in explanted lungs unusual. Its incidence ranges from 0.8% to 2% in different studies (44, 45), with adenocarcinoma being the most common histological type (7). A recent history of malignancy is considered an absolute contraindication to lung transplantation, with a 2 to 5 year cancer-free interval recommended before listing (46). To date, there are no clear consensual lines for lung cancer screening for candidates on the waiting list, with many centers using a similar approach to that recommended for the general population with annual CT scans in high-risk populations (47). However, detecting lung cancer before transplantation can be difficult due to numerous false-positive rates, and the invasive diagnosis of the tissues can be dangerous for frail patients (44).

### Testing implications

This series is the largest in the literature focusing on the development of cancer in lung grafts. To our knowledge, this is the first study to evaluate by STR technique the real origin of cancer in transplanted lungs.

The described STR technique can provide valuable information about the cellular origin of cancer after lung transplantation. The results of our study highlight that lung transplantation and the occurrence of lung cancer on a lung graft are not the only problems with the graft. This is a problem for the donor-recipient couple. This information can be used to provide advice for future donor and recipient selection, and to minimize cancer incidence. Patient counseling is important. Many patients on the waiting list were young people with no predisposition to cancer. Understanding the origin of cancer after lung transplantation can be important for both therapeutic and psychological management. Moreover, if cancer originates from the recipient, the lifestyle behaviors of transplant patients could be further changed, such as tobacco smoking.

The fact that only patients with pulmonary fibrosis and a history of smoking developed a tumor in our series where the origin is the recipient, led us to a reinforced postoperative surveillance with a double review of the scans in this population. The frequency of scans was also re-evaluated in this population with a low dose scan every 6 months (previously the frequency was annual) for the five years following lung transplantation.

The definition of the cell origin can play a major role in modifying immunosuppression. Current practice is associated with a reduction in immunosuppression, which allows immune restoration to fight malignant cells (12). However, in numerous cases of donor-derived tumors, complete remission of cancer is achieved by stopping immunosuppression followed by transplantectomy of the rejected graft (48, 49). Conventional chemotherapy carries an increased risk of infectious complications in patients with transplanted organs due to immunosuppression, but it is unlikely that cancer origin cells can modify the schedule or dosage of chemotherapy. In contrast, immunotherapy carries a substantial risk of transplant rejection in transplanted patients and may generally be avoided when rejection of the transplant could have a negative impact (50).

### Strengths and limitations

Although the prevalence in our study was similar to that of other published reports, it represents a new view of the actual prevalence of lung cancer in transplant patients and the associated biological chimera. Our study had some limitations. First, it was a monocentric study. The study was conducted over a long period, with changes in the standard of care for immunosuppression and cancer care. The follow-up time for some patients was short, with some patients being followed a few times after lung cancer development. Although we are limited by the fact that this is a monocentric cohort, the prevalence of post-transplant malignancy is consistent with the most recent published literature (7, 12) and is probably reliable given the aggressive follow-up and screening that is applied to lung transplant recipients. STR-PCR has very low sensitivity, with a rate between 1% and 5%, but can provide a good quantification capacity that is suitable for early post-transplant monitoring (15, 20). Another limitation was the low event rate of lung cancer in our cohort which made it statistically impossible to identify risk factors for the development of cancer. These limitations were also applied to describe the PDL-1 status and NGS analysis. In the era of targeted therapies and the considerable development of immunotherapy in the management of lung cancer, we aimed to gather information on the PDL-1 status of all 15 cases, together with NGS analysis. Unfortunately, this information is available only for some of the most recent cases. In addition, NGS analysis is not performed in early stage patients who have undergone surgery. We did not have enough information to provide a relevant description of these elements in our cohort. The follow-up of this cohort will continue, and we will endeavor to search for risk factors as well as to detail possible genetic particularities of these cancers in the future.

## Conclusion

Overall, our findings contribute to a better understanding of the genetic origin of lung cancer after transplantation, but further investigation is needed to clarify whether these STR loci are involved in carcinogenesis mechanisms. Three of the 14 post-transplant patients had a recipient origin for their graft lung cancer. These informative results highlight the importance of donor selection, recipient lifestyle behavior, and medical supervision after lung transplantation. However, the results of our study showed that the heterogeneity of genetic alterations using STR analysis for the 15 lung cancer specimens examined. Additional studies are required to examine the mechanisms underlying the involvement of different STR loci. A continual increase in the sample size will be necessary in future studies to clarify this discrepancy between donor and recipient malignancy origins.

## Data availability statement

The datasets presented in this study can be found in online repositories. The names of the repository/repositories and accession number(s) can be found in the article/**Supplementary Material**.

## Ethics statement

The studies involving humans were approved by the Foch Hospital Ethics Committee. The studies were conducted in accordance with local legislation and institutional requirements. All participants provided written informed consent to participate in the study. Written informed consent was obtained from all participants for publication of potentially identifiable images or data included in this article.

## Author contributions

All authors listed have made a substantial, direct, and intellectual contribution to the work and approved it for publication.
