# Supplementary material for: Donor/recipient origin of lung cancer after lung transplantation by DNA short tandem repeat analysis
Source: Front Oncol. 2023 Sep 28;13:1225538. doi: 10.3389/fonc.2023.1225538 (PMC10568626; doi:10.3389/fonc.2023.1225538)
Supplement: Supplementary file 1 [file Table_1.docx]

**Supplementary File 1: Description of the STR technique**

The AmpFlSTR™ Identifiler™ Plus PCR Amplification Kit is a short tandem repeat multiplex assay that amplifies 15 tetranucleotide repeat loci (D8S1179, D21S11, D7S820, CSF1PO, D3S1358, TH01, D13S317, D16S539, D2S1338, D19S433, vWA, TPOX, D18S51, D5S818 and FGA) and the Amelogenin gender-determining marker in a single PCR amplification.

PCR products were labeled with four fluo­rescent dyes 6-FAM™, VIC™, NED™, PET™ and detected by electrophore­sis in an 3500XL Genetic Analyzer (Applied Biosystems®), using 24 capillaries of 50cm and the POP7 polymer.

Each sample running with GeneScan 600 LIZ Sige standard (Applied Biosystems®) and positive and negative controls (Hi-Di Formamide) in each plate. These raw data were analyzed with GeneMapper™ ID Software (version 4.1.1) with Identifiler_Plus_Panels_v1 and Identifiler_Plus_Bins_v1 into the Panel Manager.

**Supplementary File 2 : STR analysis interpretation for each patient.**

| **Patients/Loci** | **D8S1179** | **D21S11** | **D7S820** | **CSF1PO** | **D3S1358** | **TH01** | **D13S317** | **D16S539** | **D2S1338** | **D19S433** | **vWA** | **TPOX** | **D18S51** | **Amelogenin** | **D5S818** | **FGA** |
| --- | --- | --- | --- | --- | --- | --- | --- | --- | --- | --- | --- | --- | --- | --- | --- | --- |
| **1-REC** | NI | NI | NI | NI | NI | NI | NI | NI | NI | NI | NI | NI | NI | NI | NI | NI |
| **1-DON** | NI | NI | NI | NI | NI | NI | NI | NI | NI | NI | NI | NI | NI | NI | NI | NI |
| **1-TUM** | NI | NI | NI | NI | NI | NI | NI | NI | NI | NI | NI | NI | NI | NI | NI | NI |
| **2-REC** | 144-144 | 202-212 | NI | NI | 124-136 | 173 | NI | NI | NI | 112-124 | 176-176 | NI | NI | 106-106 | 146-154 | NI |
| **2-DON** | NI | NI | NI | NI | NI | NI | NI | NI | NI | NI | NI | NI | NI | 106-106 | NI | NI |
| **2-TUM** | 144-144 | NI | NI | NI | 136-136 | NI | NI | NI | NI | 116-124 | NI | NI | NI | 106-106 | NI | NI |
| **3-REC** | 140-148 | NI | NI | NI | 120-124 | NI | NI | NI | NI | NI | NI | NI | NI | NI | NI | NI |
| **3-DON** | 140-144-148-152 | 198-202 | NI | NI | 120-124 | 170-178-182 | NI | NI | NI | 112-116-120-124 | 172-180 | 238-238 | NI | 106-106 | 150-150 | NI |
| **3-TUM** | 140-148 | 202-210 | NI | NI | 120-124 | 170-182 | NI | NI | NI | 112-116-120-124 | 180-184 | 230-242 | NI | 106-106 | 142-150 | NI |
| **4-REC** | 136-148 | 212-212 | NI | NI | 132-132 | 170-182 | 224-232 | 280-284 | NI | 120-126 | 164-172 | 234-242 | 292-292 | 106-106 | 146-158 | NI |
| **4-DON** | 136-148-152 | 206-212 | 267-271-275 | 322-326-330 | 120-124-132 | 170-182-186 | 224-228-232-236 | 280-280 | 318-322 | 116-120-126 | 164-172-184 | 234-242 | 284-288-292-296 | 106-106 | 146-150-158 | 232-236-248 |
| **4-TUM** | 148-152 | NI | NI | NI | 120-132 | 170-182 | 224-236 | 280-280 | NI | 116-120-126 | 164-172 | NI | NI | 106-106 | NI | NI |
| **5-REC** | 144-148 | 194-202 | NI | NI | 128-128 | 170-186 | 216-228 | 272-280 | 318-326 | 120-128 | 176-180 | 230-238 | NI | 106-112 | 132-158 | 232-236 |
| **5-DON** | NI | NI | NI | NI | NI | NI | NI | NI | NI | 120-120 | NI | NI | NI | NI | NI | NI |
| **5-TUM** | 140-156 | 208-208 | NI | NI | 108-128-132 | 170-170 | 170-170 | NI | NI | 120-120 | NI | NI | NI | 106-112 | NI | NI |
| **6-REC** | 144-152 | NI | NI | NI | 124-136 | 182-186 | NI | NI | NI | 124-130 | NI | NI | NI | 106-112 | NI | NI |
| **6-DON** | 140-144-148-152 | 204-208-212 | 275-279 | 326-326 | 120-124-128-136 | 170-182 | 228-232 | 272-280 | NI | 112-116-124-130 | 176-182-186 | 230-230 | 292-296-300-326 | 106-112 | 150-154 | 236-236 |
| **6-TUM** | 140-148-152 | NI | NI | NI | 120-124-128 | 170-182 | 228-228 | NI | NI | 116-116 | 176-176 | NI | NI | 106-112 | 150-154 | 236-236 |
| **7-REC** | 144-144 | NI | NI | NI | 120-128 | 184 | NI | NI | NI | 124-124 | 172-172 | NI | NI | 106-106 | 150-158 | NI |
| **7-DON** | 144-148 | 200-208-214 | 263-271 | 318-322 | 128-132 | 170-174 | 232-236 | 280-284 | 322-322 | 120-120 | 164-172 | 230-234 | 280-284-292 | 106-112 | 154-154 | 228-232 |
| **7-TUM** | 144-148 | 200-208 | 263-271 | 318-322 | 128-132 | 170-174 | 232-236 | 280-284 | 322-322 | 120-120 | 164-172 | 230-234 | 280-284-292 | 106-112 | 154-154 | 228-232 |
| **8-REC** | 132-156 | NI | NI | NI | 120-120 | 182-182 | NI | NI | NI | 112-120 | 176-180 | NI | NI | 106-106 | NI | NI |
| **8-DON** | 132-140 | NI | NI | NI | 120-120 | NI | NI | NI | NI | 112-116-120 | NI | NI | NI | 106-106 | NI | NI |
| **8-TUM** | 132-140 | NI | NI | NI | 120-128 | 182-182 | NI | NI | NI | 116-120 | NI | NI | NI | 106-106 | NI | NI |
| **9-REC** | NI | NI | NI | NI | NI | NI | NI | NI | NI | 116-120 | NI | NI | NI | NI | NI | NI |
| **9-DON** | 132-140-144 | NI | NI | NI | 124-128-132 | 170-182 | NI | NI | NI | 116-126 | NI | NI | NI | 106-112 | NI | NI |
| **9-TUM** | 132-140-144-148 | 200-222 | NI | NI | 124-128-132 | 170-174-182-184 | 232-232 | NI | NI | 116-120-126 | 164-172-176 | 230-230 | NI | 106-112 | 154-154 | NI |
| **10-REC** | 132-148 | 208-208 | NI | NI | 120-136 | 170-184 | NI | NI | NI | 116-122 | NI | NI | NI | 106-112 | NI | NI |
| **10-DON** | NI | NI | NI | NI | 124-124 | NI | NI | NI | NI | NI | NI | NI | NI | NI | NI | NI |
| **10-TUM1** | 132-148 | 200-208 | 263-271 | 318-318 | 120-136 | 170-184 | 216-232 | 276-284 | 338-344 | 116-122 | 172-180 | 234-242 | 288-288 | 106-112 | NI | 224-224 |
| **10-TUM2-brain** | 132-148 | 200-208 | 263-271 | 318-318 | 120-136 | 170-184 | 216-232 | 276-284 | 344-344 | 116-122 | 172-180 | 234-242 | NI | 106-112 | NI | 224-224 |
| **11-REC** | 124-132 | 200-222 | NI | NI | 120-128 | 182-186 | 224-224 | 268-280 | NI | 112-116 | 164-172 | 230-230 | 292-304 | 106-106 | 154-158 | NI |
| **11-DON** | 124-132-144-148 | 200-204-208-222 | 261-269-281-285 | 318-326 | 120-124-128-132 | 170-174-182-186 | 220-224-228 | 268-276-280 | 314-318-342 | 112-116-124-130 | 164-172-180 | 230-242 | 284-288-292-304 | 106-112 | 150-154-158 | 224-236-252 |
| **11-TUM** | 124-132-144-148 | 200-204-208-222 | 261-269-281-285 | 318-326 | 120-124-128-132 | 170-174-182-186 | 220-224-228 | 268-276-280 | 314-318-342 | 112-116-124-130 | 164-172-180 | 230-242 | 284-288-292-304 | 106-112 | 150-154-158 | 224-236-252 |
| **12-REC** | 144-152 | 204-208 | 275-275 | 332-332 | 120-120 | 178-178 | 216-232 | 268-368 | 318-330 | 120-120 | 172-176 | 226-242 | NI | 106-112 | 150-154 | 228-244 |
| **12-DON** | 128-132-144-152 | 204-208-212 | 275-279 | 318-326-330 | 120-128 | 174-178-182 | 216-232 | 268-280-284 | 318-326-330-346 | 120-124-128 | 172-176-180 | 226-230-242 | 280-284-300 | 106-106 | 146-150-154-158 | 228-232-244 |
| **12-TUM** | 128-132-144-152 | 204-208-212 | 275-279 | 318-326-330 | 120-128 | 174-178-182 | 216-232 | 268-280-284 | 318-326-330-346 | 120-124-128 | 172-176-180 | 226-230-242 | 280-284-300 | 106-106 | 146-150-154-158 | 228-232-244 |
| **13-REC** | 136-144 | NI | NI | NI | 128-132 | 182-182 | 228-232 | NI | NI | 116-120 | NI | NI | NI | 106-112 | NI | NI |
| **13-DON** | 136-140-144-148 | 198-202-208-214 | 259-267-271-275 | 318-322-326 | 120-128-132 | 170-182 | 228-232-236 | 276-280-284 | 314-330-346 | 116-120 | 164-168-172-176 | 230-238 | 280-284-308-312 | 106-112 | 150-154 | 224-234-236-244 |
| **13-TUM** | 136-140-144-148 | 198-202-208-214 | 259-267-271-275 | 318-322-326 | 120-128-132 | 170-182 | 228-232-236 | 276-280-284 | 314-330-346 | 116-120 | 164-168-172-176 | 230-238 | 280-284-308-312 | 106-112 | 150-154 | 224-234-236-244 |
| **14-REC** | 132-148 | 200-206 | NI | NI | 124-136 | 170-170 | NI | NI | NI | 112-116 | NI | NI | NI | 106-106 | NI | NI |
| **14-DON** | 132-144-148 | 200-206-210 | 267-271-279 | 318-322-326 | 124-132-136 | 170-176-182 | 220-224-232 | 276-280-284 | 310-314 | 112-116-120-124 | 168-180-188 | 230-234 | NI | 106-106 | 146-154-158 | 228-232-236-240 |
| **14-TUM1-b6** | 132-144-148 | 200-206-210 | 267-271-279 | 318-322-326 | 124-132-136 | 170-176-182 | 220-224-232 | 276-280-284 | 310-314 | 112-116-120-124 | 168-180-188 | 230-234 | NI | 106-106 | 146-154-158 | 228-232-236-240 |
| **14-TUM2-b11** | 132-144-148 | 200-206-210 | 267-271-279 | 318-322-326 | 124-132-136 | 170-176-182 | 220-224-232 | 276-280-284 | 310-314 | 112-116-120-124 | 168-180-188 | 230-234 | NI | 106-106 | 146-154-158 | 228-232-236-240 |

Rec: recipient

Don: donor

Tum: tumor
